# Book Notices

**Published:** 1880-01-20

**Authors:** 


### *BOOK NOTICES.*

PARACENTESIS OF THE PERICARDIUM, *a Consideration of the Surgical Treatment of Pericardial Effusions*, by John B. Roberts, A.M., M.D., Lecturer on Anatomy in the Philadelphia School of Anatomy, Demonstrator of Anatomy in Philadelphia Dental College, etc., etc.; with illustrations. Philadelphia, J. B. Lippincott & Co.

This is an ably-written book of 100 pages, neatly printed, and must prove very acceptable to the profession, and especially to the surgeon.

THE PHYSICIAN'S DAILY POCKFT RECORD, comprising a list of many useful memoranda, tables, etc., by S. W. Butler, M.D., fourteenth year. New and thoroughly revised, with metric osological table, etc.; edited by D. G. Brenton, Philadelphia. Published at the office of the Medical and Surgical Reporter.

MEDICAL CHEMISTRY, INCLUDING THE OUTLINES OF ORGANIC AND PHYSIOLOGICAL CHEMISTRY, based in part upon Riche's Manual [illegible]e Chemie, by C. Gilbert Wheeler, Professor of Chemistry in the University of Chicago, and formerly Professor of Organic Chemistry in the Chicago Medical College. Second and revised edition. Wm. Wood & Co. New York. 1880.

This is a volume of over four hundred pages. embracing a department of chemical study which has received too little attention. It must prove a work of great interest and value to the student, and also to the practitioner.

Professor Wheeler is the author of the following useful, able and interesting works: Derminative Mineralogy, Natural History Charts, Natural History Primmer, Catalogus Polyglottus, and Chemistry of Building Material.

THE DUTIES of the Medical Profession concerning Prostitution and Its Allied Vices Being the Oration before the Maine Medical Association at its annual meeting, 12th of June, 1878. By Frederic Henry Gerrish, M. D., Professor of Materia Medica and Therapeutics, and Lecturer on Public Health, in Bowdoin College; Instructor in Physiology and Microscopical Anatomy in the Portland School for Medical Instruction, etc.

OYSTER-SHUCKER'S CORNEITIS. (Corneitis Ostrearii.) Reprinted from Virginia Medical Monthly, February, 1879. By W. J. McDowell, M. D., Chief of Clinic to Chair of Eye and Ear Diseases University of Maryland; Attending Surgeon to Presbyterian Eye and Ear Hospital, etc., Baltimore. Read before Baltimore Med. and Surg. Society, January 16, 1879.

ON THE POSTURAL TREATMENT of Tympanites Intestinalis following Ovariotomy. By Edward W. Jenks, M D., Professor of Medical and Surgical Diseases of Women and Obstetrics, in Detroit Medical College; Fellow of the Obstetrical Society of London; Fellow of the American Gynecological Society; Honorary Member of the Cincinnati Obstetrical Society; etc., etc.

STERILITY AND ITS TREATMENT. By William H. Wathen, M. D., Clinical Lecturer on Diseases of Women and Children, Louisville Medical College; Surgeon in the Female Department, Louisville City Hospital.

ON ARTIFICIAL DISINFECTION as a Means of Preventing the Spread of Infectious Diseases. By Rev. S. H. Timins, M. A., F. G. S.; Vicar of West Malling, Kent; student of St. Thomas' Hospital, London.

OPIUM AS A TONIC AND ALTERATIVE; and its Hypodermic Use in the Debility and Amorosis sometimes consequent upon Onanism. By B. A. Pope., M. D.

THE FIRST ANNUAL REPORT of the Presbyterian Eye and Ear Charity Hospital, No. 77 East Baltimore street, Baltimore, Md. For the year ending December 2, 1878.

REMARKS ON OVARIOTOMY, with Relation of Cases and Peculiarities in Treatment. By Nathan Bozeman, M. D., New York, Surgeon to the Woman's Hospital of the State of New York, etc.

THE T EATMENT of the Genito-Urinary Organs, the Use of Electricity, Damiana, etc., etc., by John J. Caldwell, M. D., 65 North Cha'les street, Baltimore, Md.

IMPOTENCY IN WOMEN. By Ely Van De Warker, M.D., Syracuse, N. Y. New York: William Wood & Co., 27 Great Jones street. 1878.

CHLORAL INEBRIETY, read before the Kings County Medical Society, April 15, 1879. By J. B. Mattison, M. D., Brooklyn New York.

THE HAND AS A CURETTE IN POST-PARTUM HEMORRHAGE. By Henry P. C. Wilson, M. D., Baltimore, Md.

ANNUAL ADDRESS before the American Academy of Medicine, at New York, September 16th, 1879. By Lewis H. Steiner, A.M., M.D.

TRANSACTIONS of the Detroit Medical and Library Association, April, 1879. Published quarterly by the association.

STORER. TREATMENT OF STRUMOUS DISEASE.

OPHTHALMIA NEONATORUM. By Richard H. Lewis, M. D.

THE THERMANTIDOTE, *an Instrument for Preventing the Evil Effects of Heat from Paquelin's Thermo-Cautery when Operating in Deep Cavities*, by P. C. Wilson, M.D. Baltimore, Md.

ŒSOPHAGISMUS, a Typical Case of True Spasmodic Stricture of the Œsophagus Resembling Organic Stricture, Completely Cured by the Passage of a Full-size Œsophageal Sound; with Remarks on the Subject, by J. J. Henna, M.D.. Sugeon to the Out-Patient Department of Bellevue Hospital, New York, Member of the Medical Society of the County of New York, etc.

TREATMENT OF CHRONIC AURAL DISCHARGES, by Julian J. Chisolm, M.D , Professor of Eye and Ear Diseases in the University of Maryland, and Surgeon in charge of Baltimore Eye and Ear Institute ; Surgeon of Presbyterian Eye and Ear Charity Hospital, etc.

AN EXAMINATION of the Usual Signs of Dislocation of the Hip—Also AN INQUIRY into the Proper Mode of Procedure when Dislocation of the Hip is accompanied with Fracture of the Femur, by Oscar H. Allis, M.D., Surgeon to the Presbyterian Hospital. Philadelphia.

A CLINICAL CONTRIBUTION to the Study of Post-Paralytic Chorea—A CONTRIBUTION to the Study of Localized Cerebral Lesions, by E. C. Seguin, M.D., Clinical Professor of Diseases of the Mind and Nervous System, in the College of Physicians and Surgeons, New York ; President of the New York Neurological Society.

RIN[illegible]WORM IN PUBLIC INSTITUTIONS—ROSACEA, by John V. Shoemaker, A M., M.D., Lecturer on Dermatology at the Philadelphia School of Anatomy, etc.

